# Institutional Notes and News

**Published:** 1911-07-08

**Authors:** 


### INSTITUTIONAL NOTES AND NEWS.

**Worcester Infirmary and the Bishop.**

The Bishop of Worcester has written a letter regarding the finances of the Infirmary. He stated that he thought it better to withhold his formal reply until they knew the effect of Mr. Lloyd George's Bill. He thought they could appeal with more certainty when that was known. The Mayor of Worcester invited the Infirmary Committee to send four representatives to the Wesleyan Chapel on the occasion of the Coronation service. Two members of the committee volunteered to attend. The committee recently voted £5 to be spent on providing entertainment for the patients on Coronation Day, and to this sum, we learn, the Mayor has said that he would be pleased to add £10, in return for which a vote of thanks has been accorded to him. No report was issued after the committee meeting in May.

**The West London Hospital Dinner.**

In view of the review of the West London Hospital's position, which The Hospital published on April 29, page 123, and of the need for support which was there noted, it is interesting to be able to announce that at the Festival Dinner held on behalf of the hospital at the White City last week, subscriptions amounting to over five thousand guineas were announced. The Duke of Abercorn, President of the hospital, presided. There were present the Mayor of Hammersmith (Mr. Norman Shairp, who, as we noted, has supported the local memorial movement, which takes the form of naming a ward in the West London Hospital after King Edward), Lord and Lady Hamilton, Sir George Donaldson, Sir William Bull, M.P., Lord Cathcart, and the Masters of the Haberdashers', Tylers', Bricklayers', and Vintners' Companies, and the Rev. G. N. Walsh, Vicar of Hammersmith, and Mrs. Walsh. The Chairman reminded those present that the net indebtedness at the beginning of the year was £10,247. For further information concerning the hospital's affairs our readers are referred to the article already mentioned.

**New Departure at Queen Charlotte's.**

Viscount Portman presided on Friday afternoon, June 30, at a meeting at Portman Square for the inauguration of a Ladies' Association in connection with Queen Charlotte's Hospital, Marylebone Road. He explained that the object of the Association was to obtain increased support for the hospital and to spread a knowledge of the work in which it had been engaged for nearly 160 years. Most useful work might also be done by a Ladies' Association in providing linen for the mothers and infants in the hospital. Sir Samuel Scott, M.P., Chairman of the Committee of Management, referred to the increasing difficulty experienced in obtaining subscriptions for hospitals, and hoped that the Ladies' Association would be the means of increasing the income from this source. The committee wanted ladies to come and see the hospital and become interested in the work. Dr. W. J. Gow (senior physician) spoke of the valuable work done by the hospital, and urged the importance of a Ladies' Association. At other hospitals they had been most useful. A resolution for the formation of a Ladies' Association was passed. Viscountess Portman was elected President of the Association, and an Executive Committee was formed. The pages of The Hospital, for instance last week's editorial note on "Women's Work at Cardiff Infirmary," show how much such organisations are capable of doing.

